# 5-Azacytidine suppresses the incidence of abdominal aortic aneurysm through the epigenetic regulation of macrophage polarization

**DOI:** 10.1016/j.jvssci.2026.100447

**Published:** 2026-09-16

**Authors:** Go Kinoshita, Kentaro Inoue, Kohei Ueno, Yusuke Fujioka, Shinichiro Yoshino, Koichi Morisaki, Tadashi Furuyama, Tomoharu Yoshizumi

**Affiliations:** a Department of Surgery and Science, Graduate School of Medical Sciences, Kyushu University, Fukuoka, Japan; b Department of Vascular Surgery, NHO Kyushu Medical Center, Fukuoka, Japan

**Keywords:** Abdominal aortic aneurysm, Macrophage polarization, Epigenome, 5-azacytidine, DNA methyltransferase 1

## Abstract

**Objective:**

Abdominal aortic aneurysm (AAA) is a life-threatening disease characterized by chronic vascular inflammation and extracellular matrix degradation. Macrophage polarization and epigenetic regulation via DNA methylation contribute to AAA pathogenesis. This study aimed to investigate the effects of DNA methyltransferase 1 (DNMT1) inhibitor 5-azacytidine (5-aza) on AAA development and its underlying mechanisms.

**Methods:**

Apolipoprotein E knockout mice were infused with angiotensin II (Ang II) to induce AAA formation and treated with 5-aza or left untreated. Histological and immunohistochemical analyses were performed to evaluate DNA methylation status and macrophage phenotype. In vitro, RAW 264.7 macrophages were stimulated with Ang II with or without 5-aza, followed by immunostaining, reverse transcription quantitative polymerase chain reaction, and Western blotting. DNMT1 was silenced using small-interfering RNA. Promoter methylation status was evaluated by methylation-specific polymerase chain reaction. Publicly available bulk microarray datasets were analyzed to support the experimental findings.

**Results:**

5-Aza considerably reduced AAA incidence and elastin degradation in Ang II–infused apolipoprotein E knockout mice. It suppressed DNMT1 expression and global DNA methylation in the abdominal aorta and reduced M1/M2 macrophage ratio, as evidenced by decreased proinflammatory M1 macrophages. In vitro, 5-aza inhibited macrophage proliferation, migration, and matrix metalloproteinase expression. Bulk microarray analysis data supported these findings, demonstrating DNMT1 upregulation and peroxisome proliferator-activated receptor gamma (PPARγ) downregulation in Ang II treated aortas. 5-Aza reduced methylation of the *Pparg* promoter in vivo and in vitro, which was associated with increased PPARγ expression and a reduced M1/M2 macrophage ratio.

**Conclusions:**

5-Aza suppresses AAA formation by epigenetically regulating macrophage polarization through modulation of *Pparg* promoter methylation. Targeting DNA methylation represents a potential therapeutic strategy for AAA.

**Clinical relevance:**

There is currently no established pharmacological therapy for abdominal aortic aneurysm (AAA). We demonstrate that DNA methyltransferase 1 inhibition by 5-azacytidine suppresses AAA formation by reducing DNA methylation and the M1/M2 macrophage ratio via upregulation of peroxisome proliferator-activated receptor γ expression. These findings identify epigenetic regulation as a key mechanism in AAA pathogenesis. Given that 5-azacytidine is already clinically available, targeting DNA methylation may represent a potential translational strategy to inhibit AAA formation, although optimization of dosing and safety will be required.

**Article Highlights**

• **Type of Research:** Mouse model and in vitro study
• **Key Findings:** In angiotensin II-infused apolipoprotein E knockout mice, 5-azacytidine (5-aza) reduced abdominal aortic aneurysm incidence, elastin degradation, macrophage infiltration, and matrix metalloproteinase expression. In vitro, 5-aza inhibited DNA methyltransferase 1, reduced DNA methylation, and reduced M1/M2 macrophage ratio via reduced methylation of the peroxisome proliferator-activated receptor γ promoter.
• **Take Home Message:** 5-Aza suppresses abdominal aortic aneurysm formation by epigenetically regulating macrophage polarization through DNA methyltransferase 1-dependent modulation of peroxisome proliferator-activated receptor γ promoter methylation, highlighting DNA methylation as a potential therapeutic target.

Abdominal aortic aneurysm (AAA) is an atherosclerotic disease characterized by progressive aortic wall weakening, dilation, and potential rupture.^1^^,^^2^ Rupture risk increases with aneurysm size; when the aortic diameter exceeds 55 mm, the annual rupture rate reaches 3% to 15%; therefore, surgical intervention is recommended.^3^, ^4^, ^5^, ^6^ Currently, surgical repair remains the only established treatment, and no evidence-based pharmacological treatment exists for AAA.^7^^,^^8^

Early aneurysm formation is characterized by inflammatory cell infiltration and extracellular matrix degradation, with macrophages infiltrating the arterial wall at an early stage and contribute to tissue destruction.^9^, ^10^, ^11^ Classically activated macrophages (M1 macrophages) produce tissue-destructive proteins, including inducible nitric oxide synthase (iNOS) and matrix metalloproteinases (MMPs), degrade elastic fibers, and weaken the aortic wall structure.^9^^,^^12^^,^^13^ In mouse AAA models, macrophage depletion or administration of tissue-protective, alternatively activated macrophages (M2 macrophages) suppresses aneurysm formation,^2^^,^^14^ highlighting the importance of macrophage polarization.

DNA methylation is an epigenetic mechanism in which a methyl group is added to the cytosine of CpG islands in the DNA promoter region, converting it into methyl cytosine. This modification reduces the binding affinity of transcription factors to the promoter, leading to suppression of gene expression.^15^ In mammals, DNA methyltransferase 1 (DNMT1), DNMT3a, and DNMT3b maintain DNA methylation.^16^ DNMT1 adds methyl groups to newly synthesized DNA strands when the opposite strand is already methylated during DNA replication, thereby preserving the methylation pattern.^17^ Angiotensin II (Ang II) increase DNMT1 expression in mouse AAA, and enhanced DNA methylation is observed in macrophages and vascular smooth muscle cells (VSMCs) in human AAA specimens,^18^, ^19^, ^20^ suggesting that abnormal DNMT1 expression and increased DNA methylation are associated with aneurysm formation.

5-Azacytidine (5-aza) is a DNMT1 inhibitor that irreversibly binds to DNMT1 and is clinically used to treat myelodysplastic syndrome.^21^ In mouse carotid artery ligation model, 5-aza suppresses neointimal hyperplasia by inhibiting DNMT1, and modulating macrophage polarization and VSMC behavior.^22^^,^^23^

Based on these reports, we hypothesized that Ang II-induced overexpression of DNMT1 leads to increased methylation of the promoter regions of gene-regulating macrophage polarization, suppresses their expression, promotes a predominance of M1 macrophages, and drives aneurysm formation.

Herein, we aimed to elucidate the aneurysm-suppressive effects of DNMT1 inhibition by 5-aza and clarify its mechanism of action.

## Methods

### Animal experiments

#### Ethical approval

All animal experiments were approved by the Animal Care and Use Committee of Kyushu University (Approval No. A23-368-0), and all animals were handled in accordance with nationally prescribed guidelines and Animal Research Reporting of In Vivo Experiments guidelines.

#### Mouse abdominal aneurysm model

Apolipoprotein E-deficient mice (ApoE^−/−^) were bred with permission from Jackson Laboratory (Bar Harbor). Since female ApoE^−/−^ mice exhibit a low incidence of aneurysm formation in the Ang II-induced model, only male mice were used.^24^ They were maintained on a 12/12-h light/dark cycle and fed standard chow (CLEA) and water ad libitum. At the 10 to 12 weeks of age, the mice were used for experiments. Ang II (Sigma-Aldrich) was delivered at an infusion rate of 1000 ng/kg/min via subcutaneous miniosmotic pumps (Alzet) under general anesthesia, as previously described.^12^ The mice were observed daily after surgery. Tissues were harvested 28 days for histology and immunohistochemistry, and blood was collected 14 days for flow cytometry after pump implantation.

Necropsy was performed immediately after an animal’s death to confirm the cause of death before tissue harvesting. Animals with rupture findings were excluded from the histological analysis but included for AAA incidence, elastin degradation score, and mortality.

A total of 32 mice were used: 12/group for aortic tissue analyses and 4/group for flow cytometry. Allocation and exclusions are shown in, Supplementary Fig, *A* (online only).

#### Drug administration

Four days after the pump implantation, the mice were intraperitoneally administered phosphate-buffered saline (PBS; 250 μL) or 5-aza (Sigma-Aldrich) every 4 days (Fig 1, *A*).^25^ Animals were assigned to the control or 5-aza group. A fixed dose of 5-aza was selected on the basis of a previous in vivo mouse study demonstrating biological activity and tolerability with repeated administration.^25^

**Fig 1:**
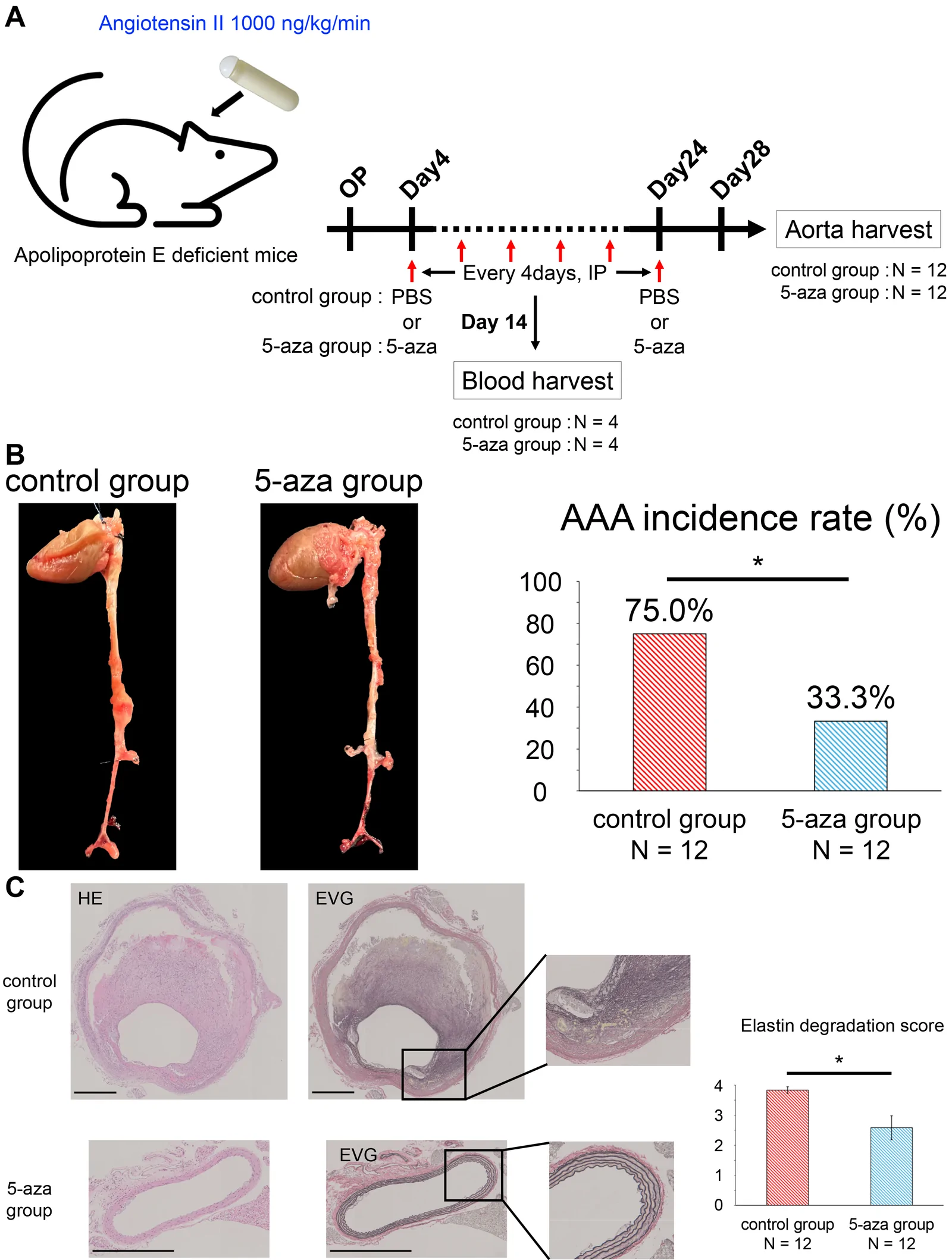

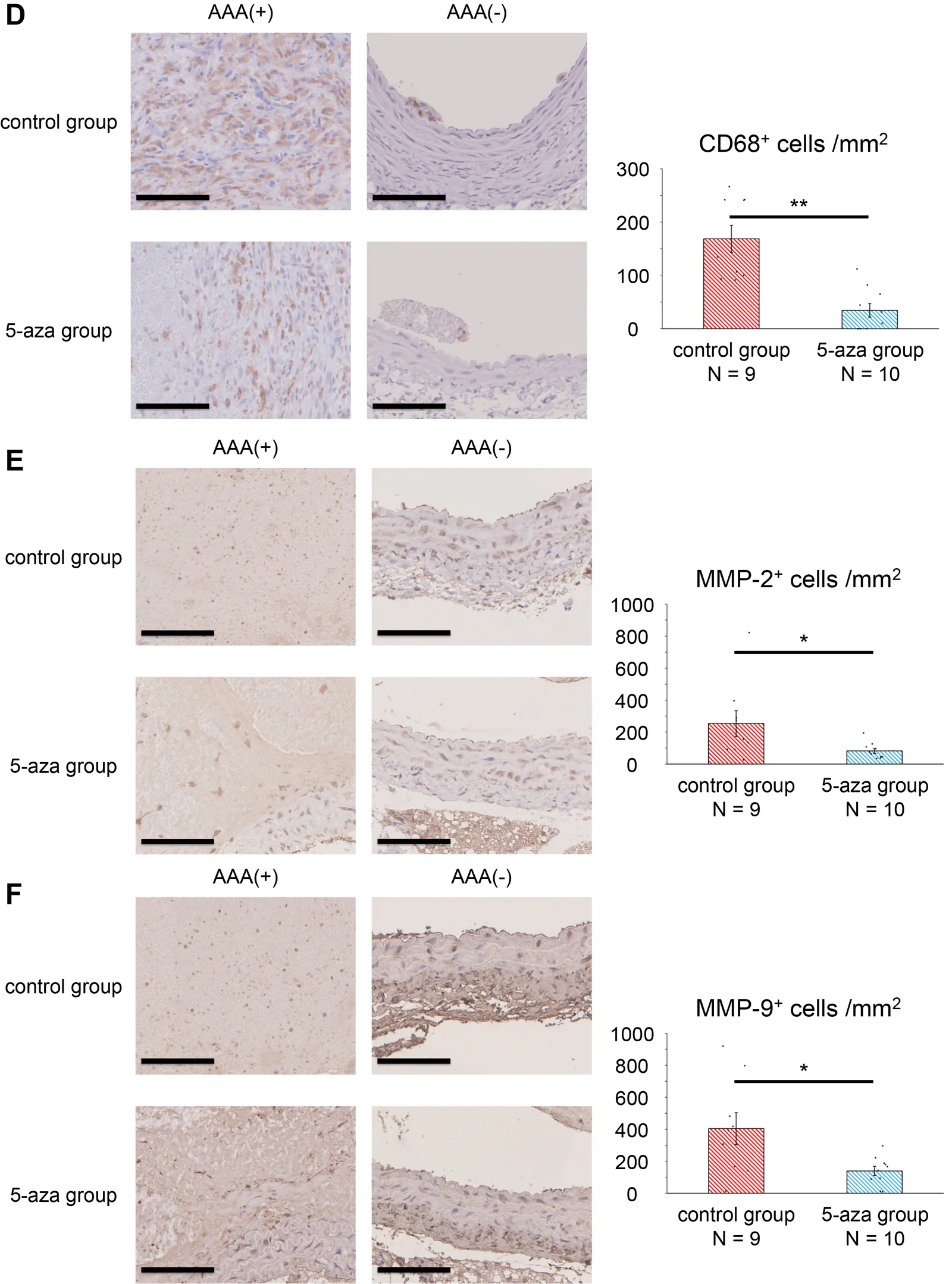
5-Azacytidine (5-aza) suppresses abdominal aortic aneurysm (*AAA*) incidence, elastin degradation, macrophage infiltration, and matrix metalloproteinase (*MMP*) expression in vivo. **A,** Experimental design. 5-Aza (5 mg/kg) or phosphate-buffered saline (*PBS*) was intraperitoneally administered every 4 days starting 4 days after angiotensin II (Ang II) pump infusion. **B,** Representative images of mice aorta. The bar graph shows the incidence of AAA (n = 12/group, χ^2^ test). **C,** Representative photomicrographs of the abdominal aortic walls stained with hematoxylin and eosin (*H&E*) and Elastica van Gieson (*EVG*). The bar graph shows the elastin degradation scores (n = 12/group, Mann-Whitney *U* test). Original magnification, ×5; Scale bar, 500 μm. **D-F,** Representative photomicrographs of the abdominal aortic wall from AAA^+^ and AAA^−^ mice stained with CD68, MMP-2, and MMP-9 antibodies. The bar graphs show the number of CD68^+^, MMP-2^+^, and MMP-9^+^ cells (n = 9-10, Mann-Whitney *U* test). Original magnification, ×40; Scale bar, 50 μm. ∗*P* < .05, ∗∗*P* < .01 indicate statistical significance.

#### Histological analyses

Histological assessments were performed using aortic tissue harvested from the descending thoracic aorta to the level of the renal artery branches 28 days after Ang II infusion. The aortas were fixed in formalin, paraffin-embedded, and sliced into 4-μm cross-sections. Hematoxylin and eosin and Elastica van Gieson staining were performed. Stained slides were scanned and analyzed using a NanoZoomer (Hamamatsu Photonics KK). The abdominal aortic diameter was calculated as external circumferential length/pi. AAA incidence was defined as a suprarenal aortic diameter >1.5 mm.^26^ The AAA incidence rate included AAA formation and death due to rupture in the mice. The areas enclosed by the luminal surface, external elastic lamina, and external circumference were measured. The elastin degradation score was graded as previously reported.^12^ For each aorta, this score was assessed on one Elastica van Gieson-stained cross-section of the suprarenal abdominal aorta at the level showing the greatest gross dilatation or at a suprarenal level in no aneurysmal aortas. Thoracic and infrarenal sections were not used for scoring.

#### Immunohistochemistry

Immunohistochemistry was performed on paraffin-embedded sections using standard protocols. Tissue sections were incubated overnight at 4 °C with the following primary antibodies: anti-CD68, anti-DNMT1, anti-5-methylcytosine (5-mC), anti-MMP-2, and anti-MMP-9 (Abcam). Subsequently, the sections were incubated with Dako EnVision + Systems-horseradish peroxidase (HRP) labeled Polymer Anti-rabbit antibody (Agilent Technologies, Inc) for 1 hour at room temperature and treated with 3,3′-diaminobenzidine. The sections were counterstained with hematoxylin and mounted on glass slides. We counted the total number of positive cells in the aortic media and defined the cell density as the number of positive cells per unit area (/mm^2^), calculated by dividing the total positive cells by the aortic wall area (mm^2^).

#### Immunofluorescence staining

Immunofluorescence staining (IFS) was performed on paraffin-embedded sections using standard protocols. Tissue sections were incubated overnight at 4 °C with anti-CD68 (Bio-Rad Laboratories, Inc) in combination with one of the following primary antibodies: anti-DNMT1, anti-5-mC, anti-iNOS (Abcam), anti-arginase-1 (Arg-1), or anti-peroxisome proliferator-activated receptor γ (PPARγ; Cell Signaling Technology). Subsequently, the sections were incubated with Alexa Fluor 488-conjugated goat anti-rat and Alexa Fluor 594-conjugated goat anti-rabbit secondary antibodies (Thermo Fisher Scientific) for 3 hours and then mounted with 4′,6-diamidino-2-phenylindole (DAPI; Thermo Fisher Scientific). The cells were observed using a fluorescence microscope (Biorevo BZ-90000, Keyence). We counted the total number of cells coexpressing CD68 with DNMT1, 5-mC, iNOS, Arg-1, or PPARγ in the abdominal aortic wall cross-section and defined the cell density as the number of double positive cells per unit area (/mm^2^), calculated by dividing the total positive cells by the aortic wall area (mm^2^). M1 macrophages were defined as CD68^+^iNOS^+^ cells, and M2 macrophages were defined as CD68^+^Arg-1^+^ cells. M1/M2 macrophage ratio was calculated.^27^ Since CD68^+^ cells were scarce in nonaneurysmal aortic walls, the M1/M2 macrophage ratio and the number of CD68^+^PPARγ^+^ cells were evaluated only in AAA-positive specimens.

#### Isolation of peripheral blood mononuclear cells from mice

On day 14 after Ang II pump implantation, whole blood was collected via cardiac puncture. Peripheral blood mononuclear cells (PBMCs) were isolated from whole blood using density gradient centrifugation with Ficoll-Paque PLUS (GE Healthcare).

#### Flow cytometry analysis

Flow cytometry was performed, as previously described.^28^ The cells were incubated with TruStain FcX (anti-mouse CD16/32) antibody to block Fc receptors. After staining with Zombie Green Fixable Viability kit to exclude dead cells, surface markers were labeled using the following fluorochrome-conjugated antibodies: anti-mouse CD45, CD11b, F4/80, CD86, and CD206 antibodies.

Macrophages were gated as CD45^+^CD11b^+^F4/80^+^ cells. M1 macrophages were defined as CD86^+^, and M2 macrophages were defined as CD206^+^ within the macrophage population. Flow cytometry was performed using a FACSLyric Flow Cytometer (BD Biosciences), and data were analyzed using FlowJo version 10.10 (BD Biosciences).

### Cell culture experiments

#### Cell culture

RAW 264.7 cells were purchased from the American Type Culture Collection (ATCC). The cells were cultured in a CO_2_ incubator at 37 °C and 5% CO_2_ in Roswell Park Memorial Institute medium 1640 (Thermo Fisher Scientific) supplemented with 10% fetal bovine serum (FBS), penicillin (100 U/mL), and streptomycin (100 μg/mL). The cells were passaged at 80% to 90% confluence and seeded into a 100-mm dish at a density of 3.0 × 10^5^ cells/mL. Cells between passages 3 and 10 were used in these experiments.

#### Drug administration

In the cell culture experiments, 24 hours after seeding, the medium was replaced as follows: control group, Dulbecco’s modified Eagle medium (DMEM; Thermo Fisher Scientific) supplemented with 0.2% FBS; Ang II group, DMEM supplemented with 0.2% FBS + Ang II 10 μM; 5-aza group, DMEM supplemented with 0.2% FBS + 5-aza 10 μM; and Ang II + 5-aza group, DMEM supplemented with 0.2% FBS + Ang II 10 μM + 5-aza 10 μM. After 24 hours, the cells were washed with PBS, and IFS or scratch assay was performed, or RNA/protein/DNA was extracted according to the following protocol. Ang II concentration was determined according to the method described by Aoyagi et al,^29^ whereas that of 5-aza was based on the method described by Kim et al.^30^

#### Small-interfering RNA-mediated knockdown of DNMT1

Cells were transfected with a nontargeting control DsiRNA (siNTC) or a DNMT1-specific DsiRNA (siDNMT1), as previously described.^31^ Both small-interfering RNAs (siRNAs) were purchased from Integrated DNA technologies (Integrated DNA Technologies). Cells were transfected using Lipofectamine RNAiMAX Transfection Reagent (Thermo Fisher Scientific) according to the manufacturer’s instructions. Total RNA was collected 48 hours after the initiation of transfection. At this point, cells were either left unstimulated or stimulated with Ang II. Accordingly, four experimental groups were established: siNTC without Ang II, siNTC with Ang II, siDNMT1 without Ang II, and siDNMT1 with Ang II. Cells were harvested for protein extraction 24 hours after Ang II stimulation.

#### IFS

Cells were seeded at a density of 1.0 × 10^6^ cells/slide in a two-well slide and chamber (Watson Co, Ltd). At 24 hours after seeding, the culture medium was replaced with 1 mL of the aforementioned medium. At 24 hours after medium change, the slides were rinsed with PBS and fixed in 1 mL formalin. After rinsing again with PBS, the slides were blocked with 10% goat serum for 1 hour at room temperature and incubated overnight at 4 °C with the following primary antibodies: anti-DNMT1, anti-5-mC, anti-iNOS (Abcam), anti-Arg-1, and anti-PPARγ (Cell Signaling Technology). Subsequently, the slides were incubated with Alexa Fluor 594-conjugated goat anti-rabbit antibody (Thermo Fisher Scientific) for 3 hours and mounted with DAPI. The cells were observed using a fluorescence microscope (Biorevo BZ-90000, Keyence). They were observed using high-power field, and images were obtained at 10 random points in the slide. The percentage of cells stained with each antibody (*red*) in the field of view was compared with the number of viable cells (DAPI-positive cells, blue).

#### Cell viability assay

Cells were seeded at a density of 2.5 × 10^5^ cells/mL in a six-well plate and incubated for 24 hours. The cells were treated under the conditions described above for 24 hours. The medium was removed, and the CellTiter-Glo 2.0 Cell Viability Assay (Promega) was performed, according to the manufacturer’s protocol.

#### Scratch assay

Cells were seeded at a density of 2.5 × 10^5^ cells/mL in a six-well plate and incubated for 24 hours. Scratches were made with a 200 μL sterile pipette tip, and the detached cells were washed away with PBS twice. After aspirating the culture medium, cells were treated under the conditions described in the “Drug administration” section of the “Cell culture experiments”, and the area between the cells was measured using ImageJ software (National Institutes of Health) at 0, 24, and 48 hours. The initial area at 0 hour was used as the reference (S_0_), and the relative change in area at 24 and 48 hours was calculated as [(S_0_–S_24_ or S_48_)/S_0_] × 100%.

#### Reverse transcription quantitative polymerase chain reaction

Total RNA from cells was extracted using Maxwell RSC simplyRNA Cells kit (Promega). RNA was reverse transcribed to cDNA using a PrimeScript first strand cDNA Synthesis Kit (Takara Bio). Reverse transcription quantitative polymerase chain reaction (RT-qPCR) was performed in duplicate using the SYBR premix EX Taq (Takara Bio) in StepOnePlus Real-Time PCR System (Thermo Fisher Scientific). Relative mRNA expressions of *Dnmt1*, *Nos2*, *Arg1*, *Mmp2*, *Mmp9*, *Pparg*, and glyceraldehyde-3-phosphate dehydrogenase (*Gapdh*) were determined using the ΔΔCt method, based on the relative expression of the target gene compared with a reference gene (*Gapdh*) and normalized to the control samples. The primers are listed in the (Supplementary Table I, online only).

#### Western blotting

Total protein from cells were extracted using radioimmunoprecipitation assay buffer (10×; Nacalai Tesque), and the protein concentration of each sample was determined using a Quick Start Bradford Protein Assay Kit 1 (Bio-Rad Laboratories, Inc). The samples were heated at 95°C for 5 minutes and electrophoresed using SuperSep Ace 5% to 12% or 10% to 20% gels (Fujifilm Wako Pure Chemical Corporation) at 20 mA for 80 minutes. The Trans-Blot Turbo Transfer System (Bio-Rad Laboratories, Inc) was used to transfer proteins onto a Trans-Blot Turbo 0.2-micron polyvinylidene difluoride membrane (Bio-Rad Laboratories, Inc). The membrane was subsequently incubated using iBind Solution Kit (Thermo Fisher Scientific) containing primary and secondary antibodies. The primary antibodies included anti-DNMT1, anti-iNOS, anti-MMP-2, anti-MMP-9, anti-GAPDH (Abcam), anti-Arg-1, and anti-PPARγ (Cell Signaling Technology) antibodies. The secondary antibodies include Goat Anti-Rabbit IgG H&L (HRP) or Goat Anti-Mouse IgG H&L (HRP; Abcam).

#### Methylation-specific PCR

Total DNA was isolated using Maxwell RSC Cultured Cells DNA Kit (Promega), according to the manufacturer’s protocol. The DNA extracted from the cells was bisulfite-converted using the Fast Bisulfite Conversion kit (Abcam). The sequence of the *Pparg* promoter region was confirmed on the Ensembl (https://asia.ensembl.org/index.html). In addition, the identification of CpG islands in the promoter region and the design of unmethylated and methylated primers were conducted on MethPrimer (https://www.urogene.org/methprimer/index1.html).

Methylation-specific PCR (MSP) was performed using EpiScope MSP kit (Takara Bio) with bisulfite-treated DNA and unmethylated and methylated primers, following the manufacturer’s instructions. The unmethylated and methylated primers for *Pparg* are listed in the Supplementary Table II (online only).

### Bioinformatics analysis of public transcriptomic data

To evaluate gene expression patterns in AAA, we analyzed publicly available bulk microarray data (GSE12591)^32^ obtained from the Gene Expression Omnibus database. This dataset includes transcriptomic profiles of abdominal aortas from ApoE^−/−^ mice infused with Ang II or saline for 28 days.

Raw expression data were downloaded and normalized using the limma package in R (version 4.5.0; R Foundation for Statistical Computing). Differentially expressed genes were identified by comparing Ang II-treated aortas with saline-treated controls. Genes with an adjusted *P* value <.05 and absolute log_2_ fold change >1 were considered significantly differentially expressed. Expression levels of PPARγ, suppressor of cytokine signaling 3, transforming growth factor-β1, interleukin-4 (IL-4), and other inflammation-related genes were specifically examined to investigate the potential involvement of methylation and macrophage polarization pathways. The volcano plot was generated using the Enhanced Volcano package.

Reagents, instruments, and antibodies are summarized in the Supplementary Table III (online only).

### Statistical analyses

Data are presented as means ± standard errors of the means. Nonparametric tests were used because of small sample sizes and non-normal distributions. Two-group and multiple-group comparisons used the Mann-Whitney *U* test and Kruskal-Wallis test with Dunn post hoc test, respectively. For two-group comparisons with fewer than five samples in either group, the exact Wilcoxon rank-sum test was used, with Cliff delta and 95% confidence intervals (CIs) reported as effect sizes.^33^^,^^34^ Categorical variables were compared using the χ^2^ test. Survival curves were analyzed using the Kaplan-Meier method and the log-rank test. All analyses were performed using JMP software (version 18.0; SAS Institute, Inc) and R version 4.5.0 for exact tests and effect-size estimation. Statistical significance was set at *P* < .05.

## Results

### 5-Aza reduces AAA incidence and elastin degradation

AAA incidence was considerably lower in the 5-aza group than the control group [5-aza group, 33.3% (4/12 mice) vs control group, 75.0% (9/12 mice; Fig 1, *B*). Rupture-related events occurred in 2/12 mice (16.7%) in the 5-aza group and 3/12 (25.0%) in controls. In the 5-aza group, one mouse died of rupture (8.3%) and one mouse survived to day 28 with a sealed rupture; all three control mice died (25.0%). Elastin degradation was markedly suppressed in the 5-aza group (Fig 1, *C*). Survival rate was 91.7% in the 5-aza group and 75.0% in the control group, with no statistically significant difference between the groups (Supplementary Fig, *B*, online only).

### 5-Aza inhibits macrophage infiltration and MMP expression in the aneurysmal wall

In vivo, we quantified the macrophages infiltration, as they accumulate from the early stages of aneurysm formation.^11^ Immunohistochemical analysis revealed a marked reduction in the number of CD68^+^ cells (macrophages) in the aortic media of the 5-aza group compared with that in the control group (Fig 1, *D*). Macrophages are a major source of MMPs, which contribute to the weakening of the aortic wall; hence, we subsequently examined their expression.^35^ The number of MMP-2^+^ or MMP-9^+^ cells was markedly lower in the 5-aza group than the control group (Fig 1, *E* and *F*). As 5-aza alters the number of macrophages and affects their functional phenotype, we evaluated macrophage polarization to investigate mechanisms.

### 5-Aza inhibits DNA methylation of macrophages and reduces the M1/M2 macrophage ratio in vivo

The number of DNMT1^+^ or 5-mC^+^ cells was considerably reduced in the 5-aza group (Fig 2, *A* and *B*). To specifically assess macrophages, double-positive CD68^+^DNMT1^+^ and CD68^+^5-mC^+^ cells were also markedly decreased in the 5-aza group (Fig 2, *C* and *D*).

**Fig 2:**
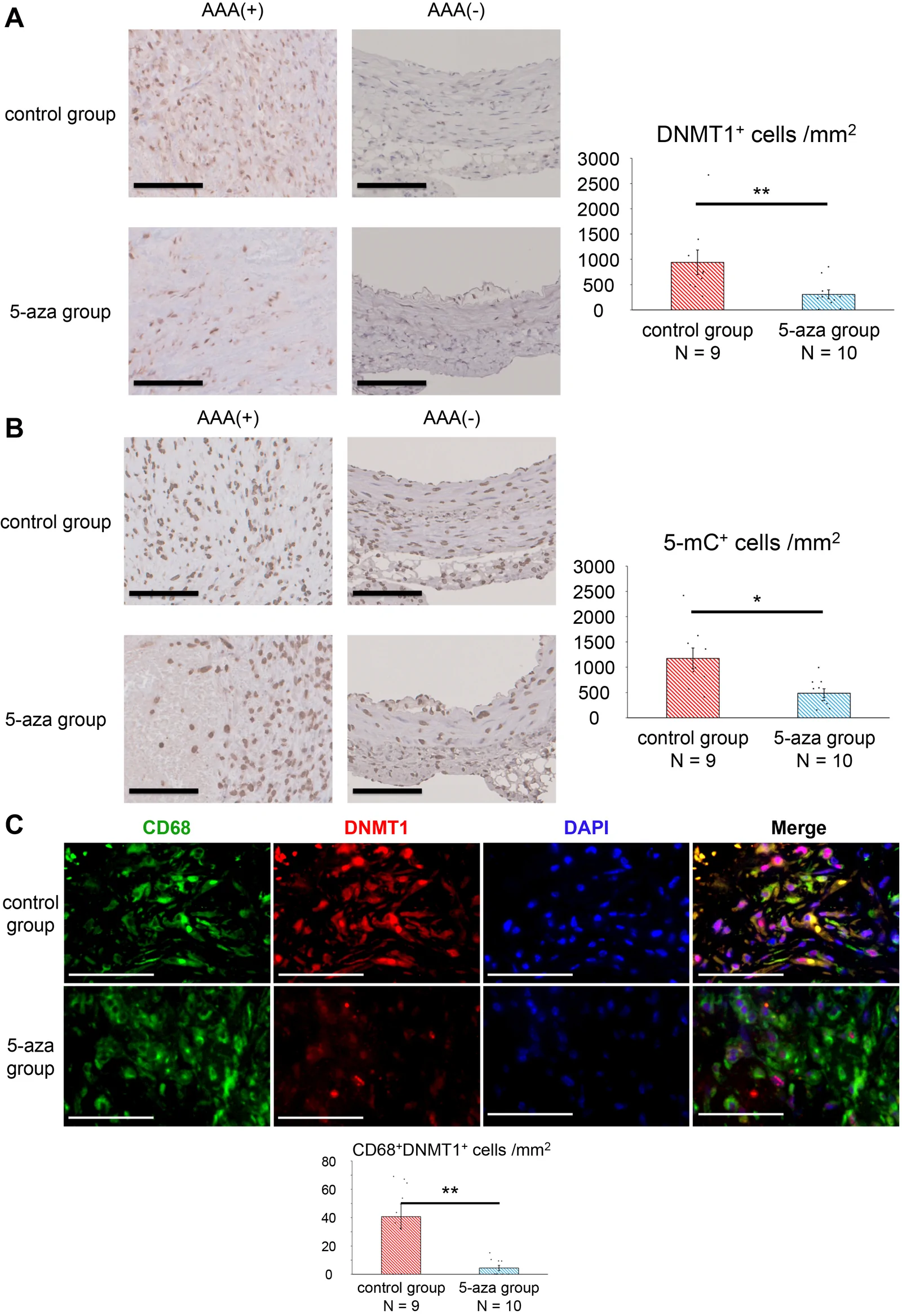

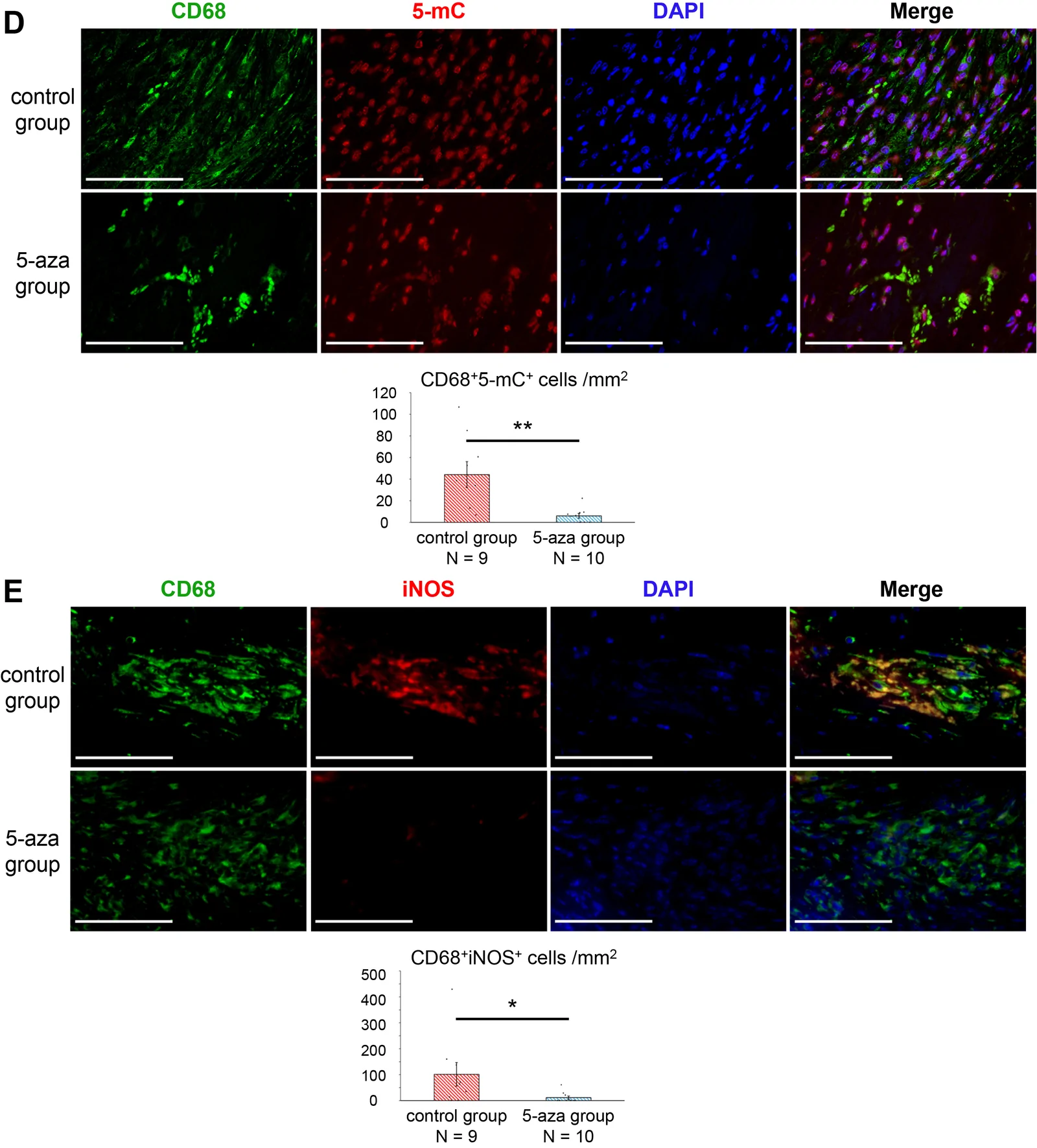

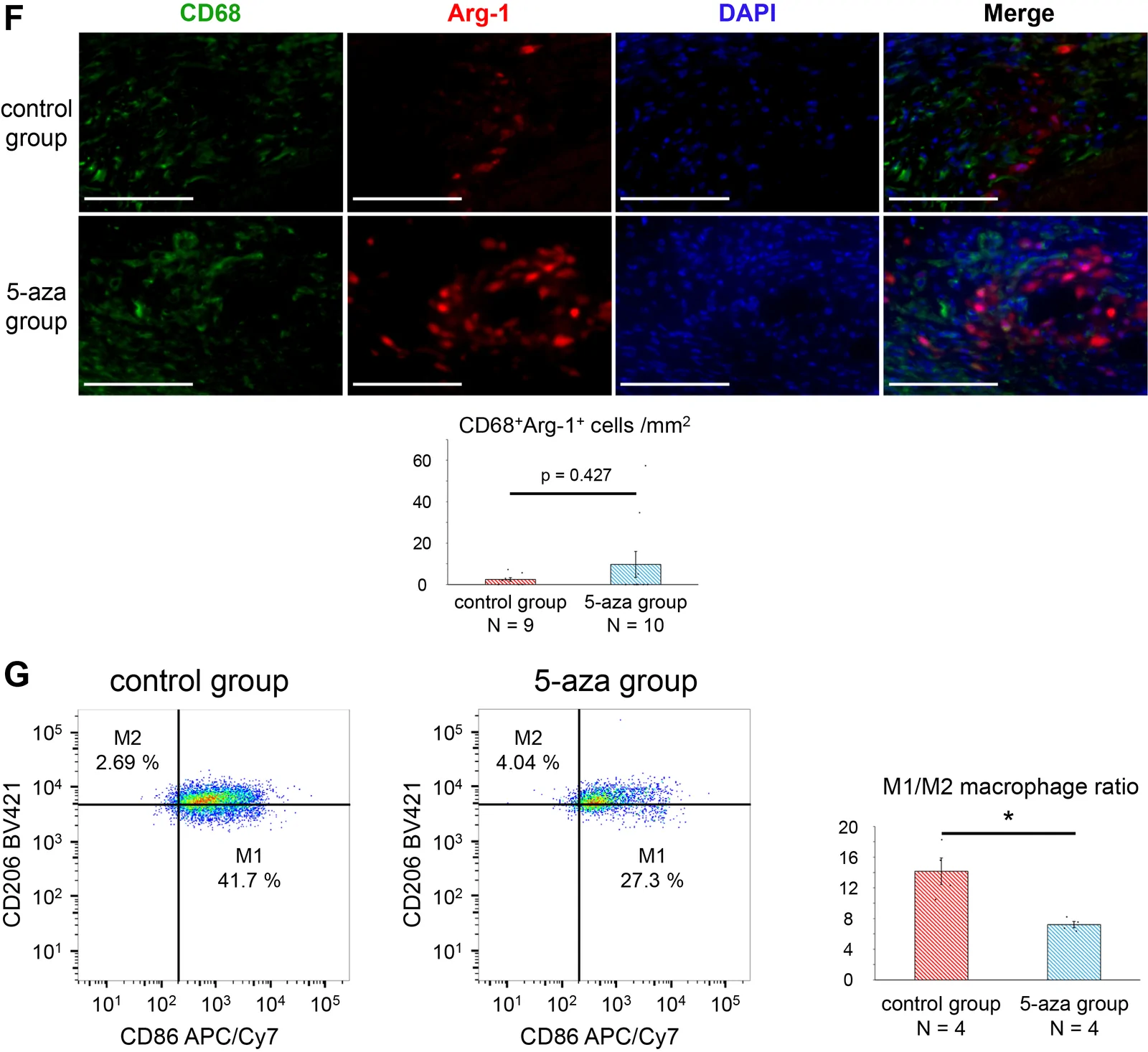
5-Azacytidine (*5-aza*) inhibits DNA methylation and shifts macrophage polarization in vivo. **A** and **B,** Representative photomicrographs of the abdominal aortic wall from abdominal aortic aneurysm (*AAA*^+^) and AAA^−^ mice stained with DNA methyltransferase 1 (*DNMT1*) and 5-methylcytosine (*5-mC*) antibodies. The bar graphs show the number of DNMT1 and 5-mC^+^ cells (n = 9-10, Mann-Whitney *U* test). Original magnification, ×40; Scale bar, 50 μm. **C** and **D,** Representative photomicrographs showing *CD68* (*green*) and DNMT1 (*red*) or 5-mC (*red*) in the AAA wall. The bar graphs show CD68^+^DNMT1^+^ and CD68^+^5-mC^+^ cells (n = 9-10, Mann-Whitney *U* test). Original magnification, ×40; Scale bar, 50 μm. **E** and **F,** Representative photomicrographs showing CD68 (*green*) and inducible nitric oxide synthase (*iNOS*; *red*) or arginase-1 (*Arg-1*; *red*) in the AAA wall. The bar graphs show CD68^+^iNOS^+^ and CD68^+^Arg-1^+^ cells (n = 9-10, Mann-Whitney *U* test). Original magnification, ×40; Scale bar, 50 μm. **G,** Representative images of flow cytometry analysis of peripheral blood mononuclear cells (PBMCs) and bar graph. The bar graph shows the M1/M2 ratio (n = 4/group, exact Wilcoxon rank-sum test). ∗*P* < .05, ∗∗*P* < .01 indicate statistical significance. *DAPI*, 4′,6-Diamidino-2-phenylindole.

Next, CD68^+^iNOS^+^ (M1) and CD68^+^Arg-1^+^ (M2) macrophages were quantified. The number of M1 macrophages was markedly reduced in the 5-aza group, whereas M2 macrophages did not differ significantly (Fig 2, *E* and *F*). Thus, the reduction in M1-associated macrophages was more pronounced than that in M2-associated macrophages. Among AAA-positive mice with available aortic tissue, M1/M2 macrophage ratio was reduced in the 5-aza group, with large effect size (exact Wilcoxon rank-sum test, *P* = .071; Cliff delta = −1.00; 95% CI, −1.00 to −0.35; Supplementary Fig, *C*, online only).^27^ Similarly, flow cytometric analysis of PBMCs also demonstrated a decrease in the M1/M2 macrophage ratio in the 5-aza group (exact Wilcoxon rank-sum test, *P* = .029; Cliff delta = −1.00; 95% CI, −1.00 to −0.64; Fig 2, *G*).

### 5-Aza suppresses macrophage proliferation, migration, and MMP expression

Given the in vivo findings, to confirm that 5-aza influenced macrophage polarization, we examined the effects of 5-aza on Ang II-stimulated macrophages in vitro. Cell viability assays showed that Ang II + 5-aza notably suppressed RAW 264.7 cell proliferation compared with cells treated with Ang II alone (Supplementary Fig, *D*, online only). Similarly, scratch assays demonstrated markedly impaired migration at both 24 and 48 hours (Fig 3, *A*). Moreover, in vitro, RT-qPCR and Western blotting (WB) showed that Ang II upregulated MMP-2 and MMP-9 expressions, whereas treatment with Ang II + 5-aza markedly reduced their expression levels (Fig 3, *B*-*E*).

**Fig 3:**
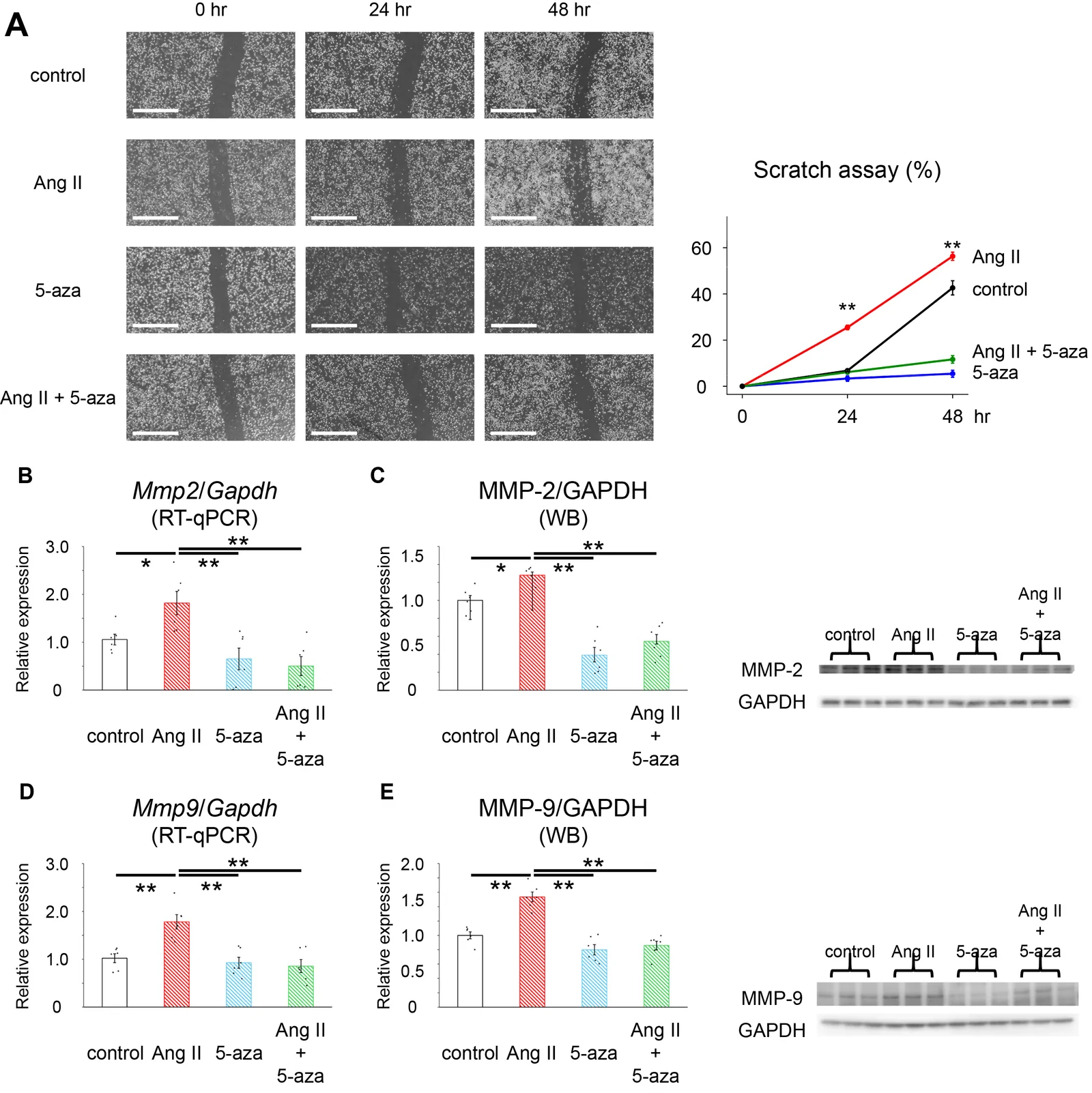
5-Azacytidine (*5-aza*) inhibits macrophage proliferation, migration, and matrix metalloproteinases (*MMP*) expression. **A,** Representative images from scratch assay. The line graph depicts the scratch assay results as the percentage change in wound area at 0, 24, and 48 hours post-treatment (n = 3, Kruskal-Wallis test with Dunn post hoc test). Original magnification, ×4; Scale bar, 1000 μm. **B,** Reverse transcription quantitative polymerase chain reaction (*RT-qPCR*) analysis of *Mmp2* expression. The bar graph shows relative mRNA expression (n = 6, Kruskal-Wallis test with Dunn post hoc test). **C,** Representative Western blotting (*WB*) analysis of MMP-2 expression. The bar graph shows relative densitometry (n = 6, Kruskal-Wallis test with Dunn post hoc test). **D,** RT-qPCR analysis of *Mmp9* expression. The bar graph shows relative mRNA expression (n = 6, Kruskal-Wallis test with Dunn post hoc test). **E,** Representative Western blotting analysis of MMP-9 expression. The bar graph shows relative densitometry (n = 6, Kruskal-Wallis test with Dunn post hoc test). ∗*P* < .05, ∗∗*P* < .01 indicate statistical significance. *Ang II*, Angiotensin II; *GAPDH*, glyceraldehyde-3-phosphate dehydrogenase.

### 5-Aza inhibits DNMT1 expression and DNA methylation in vitro

In cultured RAW 264.7, IFS showed that the Ang II + 5-aza group had a lower number of DNMT1^+^ and 5-mC^+^ cells compared with that in the Ang II group (Fig 4, *A* and *B*). These findings were supported by RT-qPCR and WB analyses, which demonstrated reduced DNMT1 expression in the Ang II + 5-aza group (Fig 4, *C* and *D*), thereby indicating that 5-aza effectively suppresses DNMT1 expression and DNA methylation in Ang II-stimulated macrophages.

**Fig 4:**
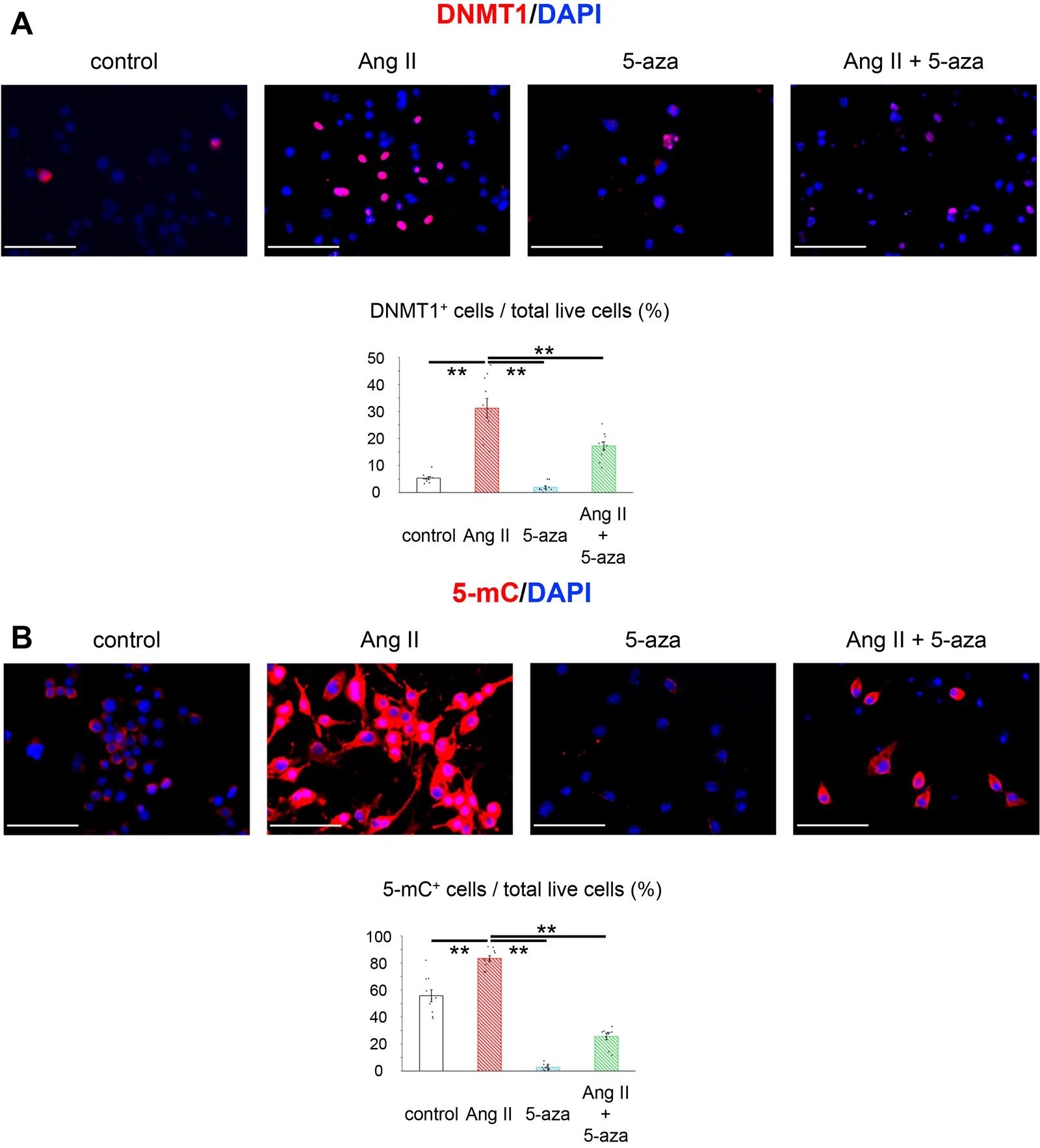

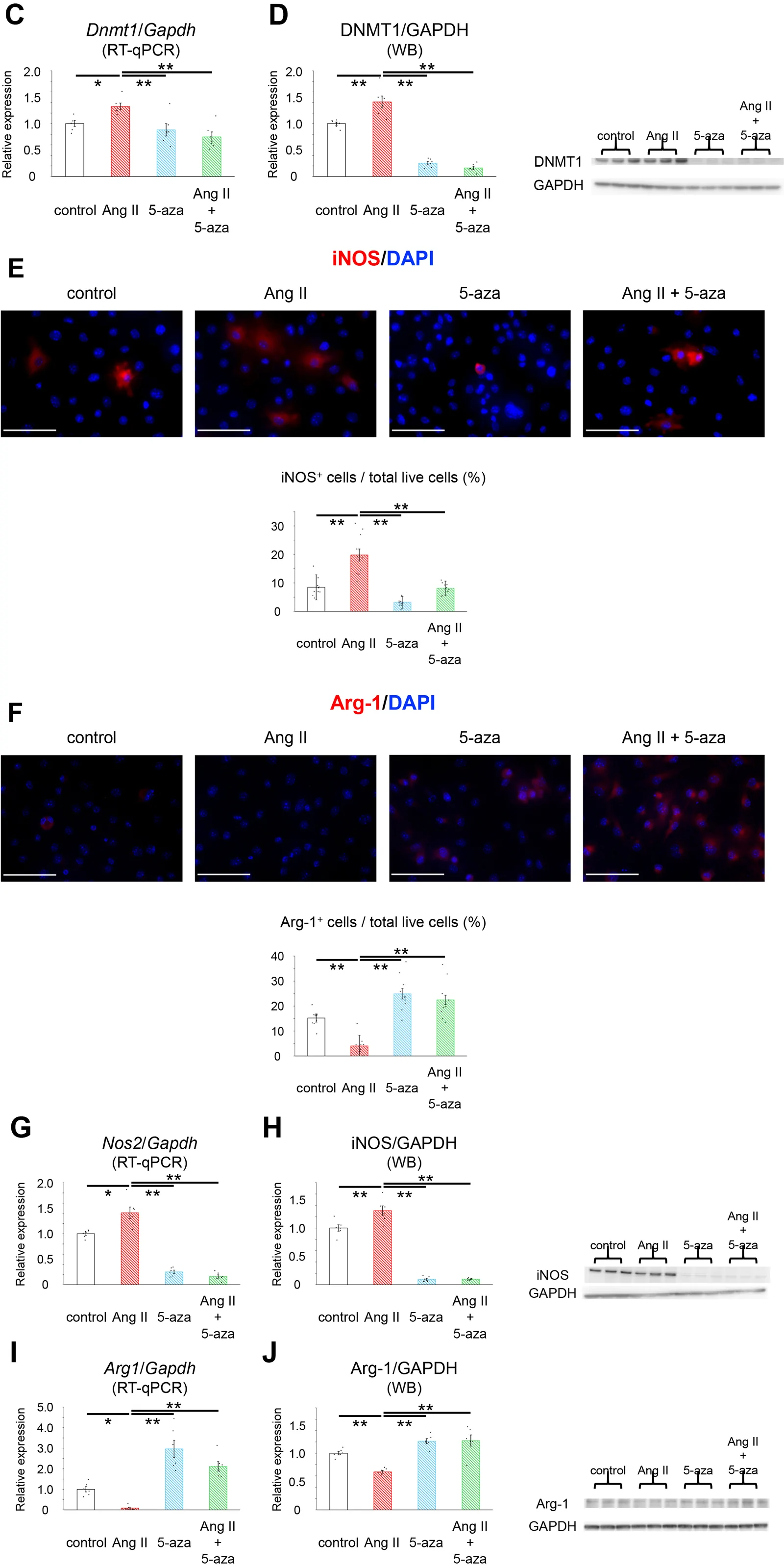
5-Azacytidine (*5-aza*) reduces DNA methyltransferase 1 (*DNMT1*) and 5-methylcytosine (*5-mC*) expression and shifts macrophage polarization in vitro. **A** and **B,** Representative photomicrographs of RAW 264.7 macrophages treated with the indicated conditions. The bar graphs show the ratio of DNMT1^+^ or 5-mC^+^ cells to live cells (n = 10, Kruskal-Wallis test with Dunn post hoc test). Original magnification, ×40; Scale bar, 50 μm. **C,** Reverse transcription quantitative polymerase chain reaction (*RT-qPCR*) analysis of *Dnmt1* expression. The bar graph shows relative mRNA expression (n = 6, Kruskal-Wallis test with Dunn post hoc test). **D,** Representative Western blotting (*WB*) of DNMT1 expression. The bar graph shows relative densitometry (n = 6, Kruskal-Wallis test with Dunn post hoc test). **E** and **F,** Representative photomicrographs of RAW 264.7 macrophages treated with the indicated conditions. The bar graph shows the ratio of inducible nitric oxide synthase (*iNOS*^+^) or arginase-1 (*Arg-1*^+^) cells to live cells (n = 10, Kruskal-Wallis test with Dunn post hoc test). Original magnification, ×40; Scale bar, 50 μm. **G,** RT-qPCR analysis of *Nos2* expression. The bar graph shows relative mRNA expression (n = 6, Kruskal-Wallis test with Dunn post hoc test). **H,** Representative Western blotting analysis of iNOS expression. The bar graph shows relative densitometry (n = 6, Kruskal-Wallis test with Dunn post hoc test). **I,** RT-qPCR analysis of *Arg1* expression. The bar graph shows relative mRNA expression (n = 6, Kruskal-Wallis test with Dunn post hoc test). **J,** Representative Western blotting analysis of Arg-1 expression. The bar graph shows relative densitometry (n = 6, Kruskal-Wallis test with Dunn post hoc test). ∗*P* < .05, ∗∗*P* < .01 indicate statistical significance. *Ang II*, Angiotensin II; *DAPI*, 4′,6-diamidino-2-phenylindole; *GAPDH*, glyceraldehyde-3-phosphate dehydrogenase.

### 5-Aza modulates macrophage polarization and related gene expression in vitro

The number of iNOS^+^ (M1) macrophages was considerably lower in the Ang II + 5-aza group compared with that in the Ang II group, whereas the number of Arg-1^+^ (M2) macrophages was markedly increased (Fig 4, *E* and *F*). This was supported by RT-qPCR and WB results, showing reduced iNOS and increased Arg-1 expressions in the Ang II + 5-aza group compared with the Ang II group (Fig 4, *G*-*J*). These findings indicate that 5-aza modulated the macrophage polarization balance by suppressing M1-associated marker expression and enhancing M2-associated marker expression in Ang II-stimulated macrophages.

### 5-Aza enhances PPARγ expression and reduces promoter methylation

To identify epigenetically regulated gene involved in macrophage polarization, we analyzed a public dataset of abdominal aortas from ApoE^−/−^ mice infused with Ang II or saline for 28 days.^32^ Among M2-associated genes, PPARγ was markedly downregulated in Ang II-treated aortas, whereas other related genes showed no significant changes (Fig 5, *A*).^36^, ^37^, ^38^, ^39^ These results prompted us to investigate PPARγ as a key regulator in the subsequent experiments. Among AAA-positive mice with available aneurysmal aortic tissue specimens, CD68+PPARγ+ cells tended to be more abundant in the 5-aza group, with large effect size (exact Wilcoxon rank-sum test, *P* = .143; Cliff delta = 0.83; 95% CI, 0.33-1.00; Fig 5, *B*). IFS, RT-qPCR, and WB analyses of macrophages confirmed markedly higher PPARγ expression in the Ang II + 5-aza group compared with that in the Ang II group (Fig 5, *C*-*E*). PPARγ expression notably differed depending on the presence or absence of 5-aza treatment; hence, we examined the methylation status of the *Pparg* promoter region. MSP analysis further demonstrated a marked reduction in methylation of the *Pparg* promoter region in the Ang II + 5-aza group compared with that in the Ang II group (Fig 5, *F*). These findings suggest that 5-aza promotes M2 macrophage polarization through modulation of *Pparg* promoter methylation.

**Fig 5:**
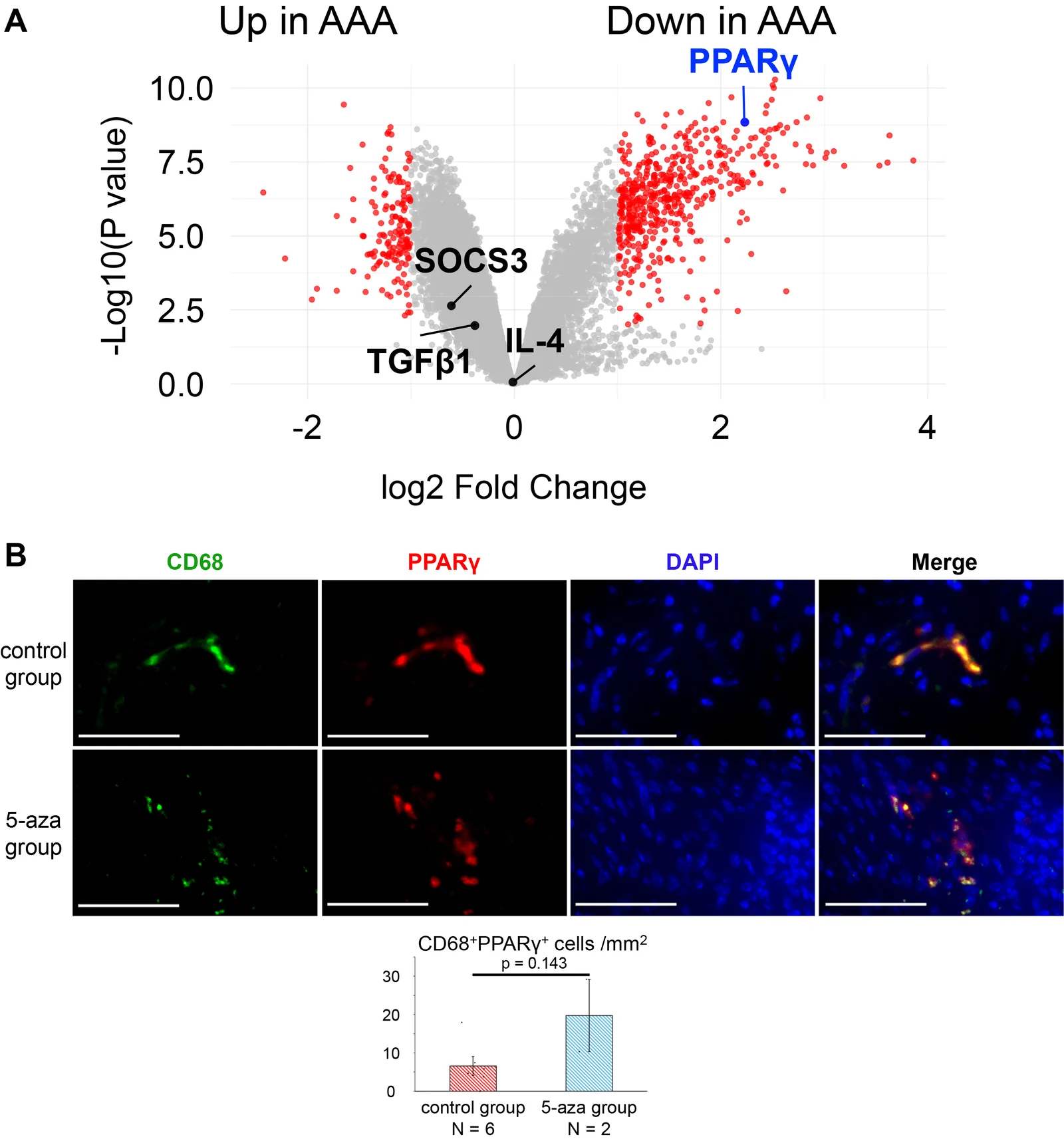

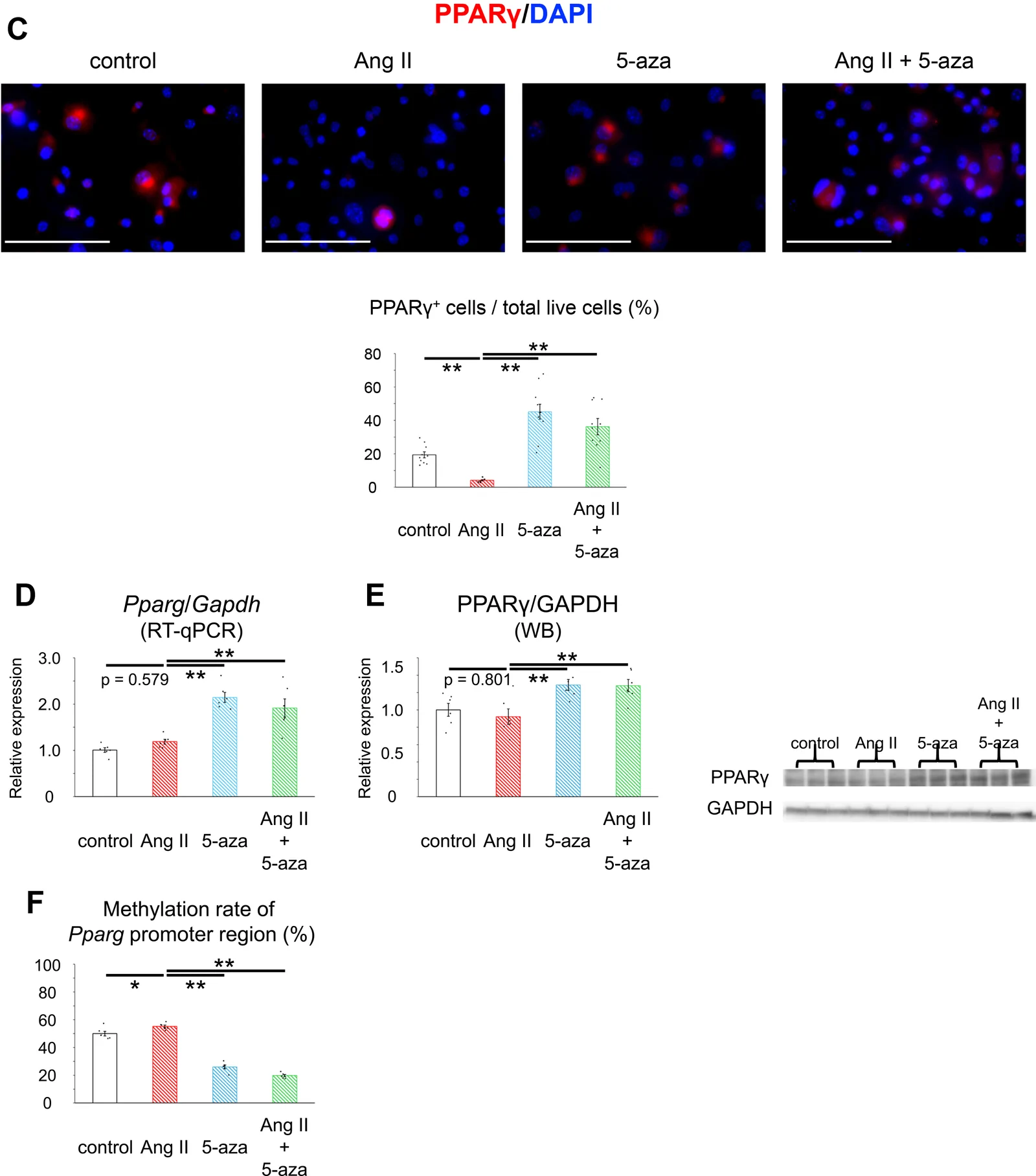
5-Azacytidine (*5-aza*) promotes peroxisome proliferator-activated receptor gamma (*PPARγ*) expression by demethylating its promoter. **A,** The volcano plot of differentially expressed mRNAs in GSE12591 microarray data. **B,** Representative photomicrographs of aortic sections from mice with abdominal aortic aneurysm (*AAA*) showing CD68^+^PPARγ^+^ macrophages (n = 2-6, exact Wilcoxon rank-sum test). Original magnification, ×40; Scale bar, 50 μm. **C,** Representative photomicrographs of RAW 264.7 macrophages treated with the indicated conditions. The bar graph shows the ratio of PPARγ^+^ cells to live cells (n = 10, Kruskal-Wallis test with Dunn post hoc test). Original magnification, ×40; Scale bar, 50 μm. **D,** Reverse transcription quantitative polymerase chain reaction (*RT-qPCR*) analysis of *Pparg* expression. The bar graph shows relative mRNA expression (n = 6, Kruskal-Wallis test with Dunn post hoc test). **E,** Representative Western blotting (*WB*) analysis of PPARγ expression. The bar graph shows relative densitometry (n = 6, Kruskal-Wallis test with Dunn post hoc test). **F,** Methylation-specific PCR (MSP) of the *Pparg* promoter. The bar graph shows relative densitometry (n = 6, Kruskal-Wallis test with Dunn post hoc test). ∗*P* < .05, ∗∗*P* < .01 indicate statistical significance. *Ang II*, Angiotensin II; *DAPI*, 4′,6-diamidino-2-phenylindole; *GAPDH*, glyceraldehyde-3-phosphate dehydrogenase; *SOCS3*, Suppressor of cytokine signaling 3; *TGF-β1*, transforming growth factor-β1.

### DNMT1 knockdown phenocopies the effects of 5-aza on Ang II-stimulated macrophages

To determine whether these effects were mediated by DNMT1 inhibition, we examined DNMT1 knockdown using siRNA. siDNMT1 reduced DNMT1 mRNA expression compared with siNTC (Fig 6, *A*). Compared with siNTC + Ang II group, siDNMT1 or siDNMT1 + Ang II significantly reduced DNMT1 and iNOS expression while increasing Arg-1 expression (Fig 6, *B*-*D*). PPARγ expression was significantly higher in the siDNMT1 + Ang II group than in the siNTC + Ang II group (Fig 6, *E*).

**Fig 6:**
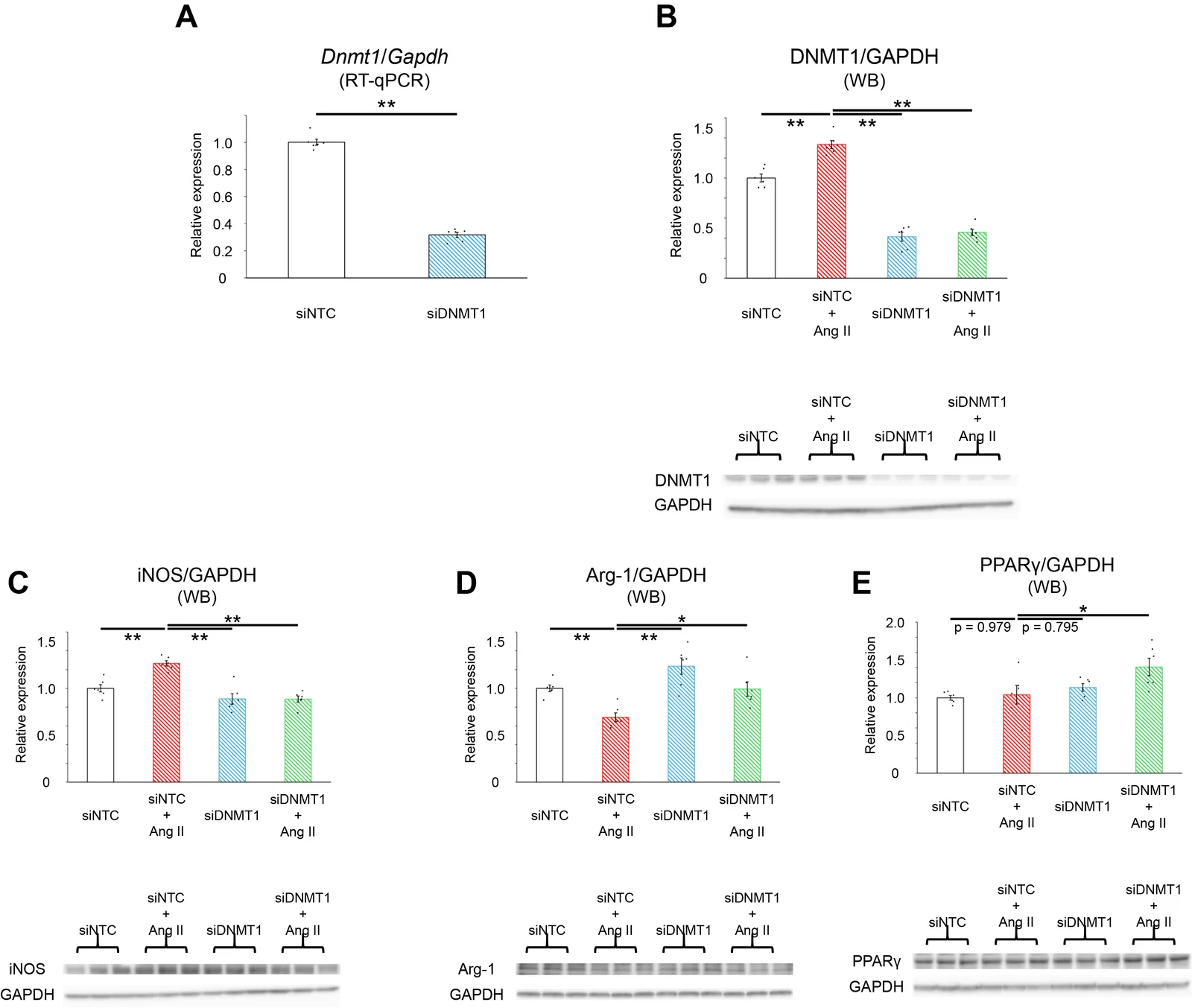
Knockdown of DNA methyltransferase 1 (*DNMT1*) phenocopies the effects of 5-azacytidine (*5-aza*) on angiotensin II (*Ang II*)-stimulated macrophages. RAW 264.7 macrophages were transfected with nontargeting control DsiRNA (*siNTC*) or DNMT1-specific DsiRNA (*siDNMT1*) and stimulated with Ang II as indicated. **A,** Reverse transcription quantitative polymerase chain reaction (*RT-qPCR*) analysis of *Dnmt1* expression 48 hours after the initiation of transfection. The bar graph shows relative mRNA expression (n = 6, Mann-Whitney *U* test). **B-E,** Representative western blotting analysis of DNMT1, inducible nitric oxide synthase (*iNOS*), arginase-1 (*Arg-1*), and peroxisome proliferator-activated receptor gamma (*PPARγ*) expression. The bar graph shows relative densitometry (n = 6, Kruskal-Wallis test with Dunn post hoc test). ∗*P* < .05, ∗∗*P* < .01 indicate statistical significance. *GAPDH*, glyceraldehyde-3-phosphate dehydrogenase; *WB*, Western blotting.

## Discussion

Herein, we aimed to investigate whether 5-aza inhibits AAA formation via the epigenetic modulation of macrophage polarization. Using an Ang II infused ApoE^−/−^ mouse model, we demonstrated that 5-aza suppressed AAA incidence (Fig 1, *B*). Histological analysis showed that 5-aza reduced the infiltration of macrophages with high levels of DNA methylation markers (DNMT1 and 5-mC) in the aneurysmal wall and reduced the M1/M2 macrophage ratio (Fig 2 and Supplementary Fig, *C*). Flow cytometry analysis showed that 5-aza reduced the M1/M2 macrophage ratio in the PBMCs (Fig 2, *G*). These findings indicate that an M1-dominant macrophage balance is associated with AAA formation and that 5-aza modulates this balance. Our data do not support direct conversion of macrophages from M1 to M2 phenotype. Rather, 5-aza reduced overall macrophage accumulation, with a greater reduction in M1-associated macrophages and relative preservation of M2-associated macrophages.

Both DNMT1 genetic deletion and pharmacological inhibition by 5-aza promote M2 macrophage polarization. In a Western diet-induced nonalcoholic fatty liver disease mouse model, hepatic Kupffer cells exhibited increased DNMT1 expression, promoter region hypermethylation of autophagy-related genes, impaired autophagy, and predominance of M1 phenotype. However, 5-aza reduces the methylation of autophagy-related genes and promotes M2 polarization.^39^ Similarly, in an obese mouse model, DNMT1 upregulation in adipose tissue macrophages leads to *Pparg* promoter methylation, suppressed PPARγ expression, and M1 polarization, whereas 5-aza treatment promotes M2 polarization.^40^ Consistent with these reports, DNMT1 silencing in Ang II-stimulated macrophages phenocopied the effects of 5-aza, suppressing iNOS expression while enhancing Arg-1 and PPARγ expression (Fig 6, *C*-*E*). Beyond macrophages, aberrant DNA methylation is implicated in other vascular compartments, including VSMCs,^41^ endothelial cells,^42^ and adventitial fibroblasts.^43^ In VSMCs, pharmacologic inhibition of DNMT1 with 5-aza reduces MMP-2 and MMP-9 expression, thereby limiting elastin degradation.^41^ In endothelial cells, DNA methylation suppresses endothelial nitric oxide synthase expression. Treatment with 5-aza restores endothelial nitric oxide synthase levels, leading to the recovery of endothelial function and anti-inflammatory activity.^42^ In fibroblasts, DNMT1 upregulation drives DNA hypermethylation and fibrotic progression, which are attenuated by 5-aza.^43^ Although our data focus on the macrophage DNMT1-PPARγ axis, potential parallel effects on VSMCs, endothelium, and adventitia cannot be excluded.

We focused on PPARγ as a target molecule whose expression is suppressed by DNA methylation, which in turn regulates macrophage polarization. PPARγ is a nuclear receptor type transcription factor that is stimulated by IL-4 or IL-13 via the signal transducer and activator of transcription 6 signaling pathway to promote the transcription of Arg-1 while suppressing iNOS transcription.^44^ This process shifts macrophage polarization to favor the M2 phenotype.^38^ Reduced expression of PPARγ shifts macrophages toward the M1 phenotype, contributing to aneurysm formation. VSMC-specific PPARγ deletion exacerbated aortic dilatation and elastin degradation in a calcium chloride-induced AAA model, supporting a protective role of PPARγ across models.^45^ However, DNMT1 inhibition requires validation in additional AAA models. In addition, analyses of publicly available gene expression datasets comparing human AAA with healthy aortic tissue have also demonstrated reduced PPARγ expression in aneurysmal tissue, attributed to its mRNA regulation by the miRNA-circRNA axis, which further contributes to inflammation and aneurysm formation.^46^ The modest increase in *Pparg* promoter methylation after 24 hours of Ang II stimulation may have been insufficient to reduce total PPARγ expression. Nevertheless, 5-aza-induced promoter demethylation was accompanied by increased *Pparg* mRNA and protein expression.

Although PPARγ is central to our model, other macrophage polarization pathways may also contribute to AAA pathogenesis. Previous studies suggest that inflammatory pathways such as Toll-like receptor 4-nuclear factor-κB can be regulated by DNMT1-dependent DNA methylation. The involvement of these additional pathways under Ang II stimulation with or without 5-aza warrants further investigation.^47^^,^^48^

### Limitations

This study has some limitations. First, although 5-aza altered DNA methylation and macrophage polarization, we did not directly assess whether these effects are exclusively mediated through DNMT1 and PPARγ. Because 5-aza was not directly compared with other DNMT inhibitors or PPARγ-targeting agents, its relative efficacy and therapeutic advantage for small AAA remain unclear. Second, the Ang II-infused ApoE^−/−^ mouse model may not fully recapitulate the complexity of human AAA. Third, as phenotypically switched VSMCs can express CD68, some CD68^+^DNMT1^+^ cells may not represent bona fide macrophages. Effects of 5-aza and PPARγ signaling on VSMC phenotype therefore cannot be excluded. Direct, cell type-specific bisulfite sequencing was beyond the scope of this study. Finally, the temporal dynamics and reversibility of DNMT1 inhibition-mediated macrophage polarization during aneurysm progression were not evaluated and warrant further investigation. Although the dose used was previously tolerated in mice, formal dose-response and safety analyses were not performed. Further studies are required to establish the optimal therapeutic range for AAA prevention.

## Conclusions

Collectively, these results highlight the importance of epigenetic modulation of macrophage polarization in AAA pathogenesis. Our study identifies DNA methylation of the *Pparg* promoter as a previously unrecognized and therapeutically targetable mechanism contributing to macrophage-mediated inflammation and extracellular matrix degradation in the aortic wall. 5-Aza is associated with reduced DNA methylation in the promoter region of PPARγ, accompanied by increased PPARγ expression and reduced M1/M2 macrophage ratio. These findings underscore the therapeutic potential of targeting DNA methylation pathways, such as with 5-aza, for AAA prevention and treatment.

## Author Contributions

Conception and design: GK, KI, KM

Analysis and interpretation: GK, KU, YF, SY, KM, TF, TY

Data collection: GK, KI

Writing the article: GK, KI, KM

Critical revision of the article: KI, KU, YF, SY, KM, TF, TY

Final approval of the article: GK, KI, KU, YF, SY, KM, TF, TY

Statistical analysis: GK, KI, KM

Obtained funding: Not applicable

Overall responsibility: GK

## Funding

None.

## Disclosures

The authors declare that they have no conflicts of interest.

## Appendix (online only)

Supplementary Tables I-III (online only)
