# Supplementary material for: 5-Azacytidine suppresses the incidence of abdominal aortic aneurysm through the epigenetic regulation of macrophage polarization
Source: JVS Vasc Sci. 2026 Sep 16;7:100447. doi: 10.1016/j.jvssci.2026.100447 (PMC13734897; doi:10.1016/j.jvssci.2026.100447)
Supplement: Supplementary file 1 [file mmc1.docx]

**Supplemental Material**

**Supplemental Table I.** **RT-qPCR primers**

| **Primer name** | **Forward primer** | **Reverse primer** |
| --- | --- | --- |
| *Dnmt1* | 5′- GGACAAGGAGAATGCCATGAAGC -3′ | 5′- TTACTCCGTCCAGTGCCACCAA -3′ |
| *Mmp2* | 5′- ATCAGGAGGAATGCCATCCC -3′ | 5′- GCTGGCATCATCATAGGATC -3′ |
| *Mmp9* | 5′- TGGGCTACGTGACCTATGACAT -3′ | 5′- CGGCAAGTCTTCCGAGTAGTTT -3′ |
| *Nos2* | 5′- GAGACAGGGAAGTCTGAAGCAC -3′ | 5′- CCAGCAGTAGTTGCTCCTCTTC -3′ |
| *Arg1* | 5′- CATTGGCTTGCGAGACGTAGAC -3′ | 5′- GCTGAAGGTCTCTTCCATCACC -3′ |
| *Pparg* | 5′- GTACTGTCGGTTTCAGAAGTGCC -3′ | 5′- ATCTCCGCCAACAGCTTCTCCT -3′ |
| *Gapdh* | 5′- CATCACTGCCACCCAGAAGACTG -3′ | 5′- ATGCCAGTGAGCTTCCCGTTCAG -3′ |

**Supplemental Table II.** **MSP primers**

| **Primer name** | **Forward primer** | **Reverse primer** |
| --- | --- | --- |
| Methylated *Pparg* | 5′- GTTTTCGGTTTTATTTTGGGTATC -3′ | 5′- ACGACGACTTAACGAATACTATACGT -3′ |
| Unmethylated *Pparg* | 5′-GGTTTTTGGTTTTATTTTGGGTATT -3′ | 5′- AAAACAACAACTTAACAAATACTATACATC -3′ |

**Supplemental Table III.** **Material list**

| **Animal experiments** |  |  |  |
| --- | --- | --- | --- |
| **Material** | **Catalog number** | **Dilution** | **Manufacturer** |
| Osmotic pumps | Model 2004 |  | Alzet, Cupertino, CA, USA |
| Angiotensin II human | A9525 | 6 mg/mL | Sigma-Aldrich, St. Louis, MO, USA |
| 5-Azacytidine | A2385 | 5 mg/kg | Sigma-Aldrich, St. Louis, MO, USA |
| Anti-CD68 antibody | ab125212 | IHC: 1:100 | Abcam, Cambridge, UK |
| Anti-DNMT1 antibody | ab188453 | IHC: 1:100  IFS: 1:100 | Abcam, Cambridge, UK |
| Anti-5-mC antibody | ab214727 | IHC: 1:100  IFS: 1:100 | Abcam, Cambridge, UK |
| Anti-MMP-2 antibody | ab97779 | IHC: 1:100 | Abcam, Cambridge, UK |
| Anti-MMP-9 antibody | ab38898 | IHC: 1:100 | Abcam, Cambridge, UK |
| Dako EnVision+ Systems HRP-labelled polymer anti-rabbit | K4003 |  | Agilent Technologies, Inc., Santa Clara, CA, USA |
| Anti-CD68 antibody | MCA1957 | IFS: 1:100 | Bio-Rad Laboratories, Inc., Hercules, CA, USA |
| Anti-iNOS antibody | ab15323 | IHC: 1:100  IFS: 1:100 | Abcam, Cambridge, UK |
| Anti-Arginase-1 antibody | 93668S | IHC: 1:100  IFS: 1:100 | Cell Signaling Technology, Danvers, MA, USA |
| Anti-PPARγ antibody | 2435S | IFS: 1:100 | Cell Signaling Technology, Danvers, MA, USA |
| Alexa Fluor 488-conjugated goat anti-rat antibody | A11006 | IFS: 1:500 | Thermo Fisher Scientific, Waltham, MA, USA |
| Alexa Fluor 594-conjugated goat anti-rabbit antibody | A11012 | IFS: 1:500 | Thermo Fisher Scientific, Waltham, MA, USA |
| 4′,6-diamidino-2-phenylindole (DAPI) | S36920 |  | Thermo Fisher Scientific, Waltham, MA, USA |
| Ficoll-Paque™ PLUS | 17-1440-02 |  | GE Healthcare, Chicago, IL, USA |
| TruStain FcX™ (anti-mouse CD16/32) Antibody | 101320 | 2.0 µL/1.0×10^6^ cells | BioLegend, San Diego, CA, USA |
| Zombie Green™ Fixable Viability Kit | 423111 | 100 µL/1.0×10^6^ cells | BioLegend, San Diego, CA, USA |
| Brilliant Violet 510™ anti-mouse CD45 antibody | 103137 | 5 µL/1.0×10^6^ cells | BioLegend, San Diego, CA, USA |
| APC anti-mouse/human CD11b Antibody | 101212 | 1.25 µL/1.0×10^6^ cells | BioLegend, San Diego, CA, USA |
| PE anti-mouse F4/80 Antibody | 123110 | 5 µL/1.0×10^6^ cells | BioLegend, San Diego, CA, USA |
| Brilliant Violet 421™ anti-mouse CD206 (MMR) Antibody | 141717 | 5 µL/1.0×10^6^ cells | BioLegend, San Diego, CA, USA |
| APC/Cyanine7 anti-mouse CD86 Antibody | 105030 | 1.25 µL/1.0×10^6^ cells | BioLegend, San Diego, CA, USA |
|  |  |  |  |
| **Cell culture experiments** |  |  |  |
| **Material** | **Catalog number** | **Dilution** | **Manufacturer** |
| RAW 264.7 cells | TIB-71^TM^ |  | ATCC, Manassas, VA, USA |
| Roswell Park Memorial Institute medium 1640 | 11875093 |  | Thermo Fisher Scientific, Waltham, MA, USA |
| Dulbecco’s modified Eagle medium | 11995065 |  | Thermo Fisher Scientific, Waltham, MA, USA |
| Angiotensin II human | A9525 | 10 µmol/L | Sigma-Aldrich, St. Louis, MO, USA |
| 5-Azacytidine | A2385 | 10 µmol/L | Sigma-Aldrich, St. Louis, MO, USA |
| non-targeting control DsiRNA | 51-01-14-03 |  | Integrated DNA Technologies, Coralville, IA, USA |
| DNMT1-specific DsiRNA | mm.Ri.dnmt1.13.3 |  | Integrated DNA Technologies, Coralville, IA, USA |
| Lipofectamine™ RNAiMAX Transfection Reagent | 13778150 |  | Thermo Fisher Scientific, Waltham, MA, USA |
| two-well slide and chamber | 192-002 |  | Watson Co., Ltd., Tokyo, Japan |
| Anti-DNMT1 antibody | ab188453 | IFS: 1:200  WB: 1:1000 | Abcam, Cambridge, UK |
| Anti-5-mC antibody | ab214727 | IFS: 1:200 | Abcam, Cambridge, UK |
| Anti-iNOS antibody | ab15323 | IFS: 1:200  WB: 1:1000 | Abcam, Cambridge, UK |
| Anti-Arginase-1 antibody | 93668S | IFS: 1:200  WB: 1:500 | Cell Signaling Technology, Danvers, MA, USA |
| Anti-PPARγ antibody | 2435S | IFS: 1:100  WB: 1:500 | Cell Signaling Technology, Danvers, MA, USA |
| Alexa Fluor 594-conjugated goat anti-rabbit antibody | A11012 | IFS: 1:500 | Thermo Fisher Scientific, Waltham, MA, USA |
| CellTiter-Glo® 2.0 Cell Viability Assay | G9241 |  | Promega, Madison, WI, USA |
| Maxwell® RSC simplyRNA Cells Kit | AS1390 |  | Promega, Madison, WI, USA |
| PrimeScript™ 1st strand cDNA Synthesis Kit | 6110A |  | Takara Bio, Shiga, Japan |
| SYBR premix EX Taq™ | RR041A |  | Takara Bio, Shiga, Japan |
| RIPA buffer (10×) | 08714-04 |  | Nacalai Tesque, Kyoto, Japan |
| Bio-Rad Protein Assay Kit, Quick Start™ Bradford Kit 1 | 5000201 |  | Bio-Rad Laboratories, Inc., Hercules, CA, USA |
| SuperSep Ace 5-12% | 192-15201 |  | Fujifilm Wako Pure Chemical Corporation, Osaka, Japan |
| SuperSep Ace 10-20% | 198-15041 |  | Fujifilm Wako Pure Chemical Corporation, Osaka, Japan |
| Trans-Blot Turbo 0.2-micron PVDF Membrane | 1704156 |  | Bio-Rad Laboratories, Inc., Hercules, CA, USA |
| iBind™ Solution Kit | SLF1020 |  | Thermo Fisher Scientific, Waltham, MA, USA |
| Anti-MMP-2 antibody | ab97779 | WB: 1:1000 | Abcam, Cambridge, UK |
| Anti-MMP-9 antibody | ab38898 | WB: 1:1000 | Abcam, Cambridge, UK |
| anti-GAPDH antibody | ab8245 | WB: 1:5000 | Abcam, Cambridge, UK |
| Goat Anti-Rabbit IgG H&L (HRP) | ab6721 | WB: 1:10000 | Abcam, Cambridge, UK |
| Goat Anti-Mouse IgG H&L (HRP) | ab6789 | WB: 1:10000 | Abcam, Cambridge, UK |
| Maxwell® RSC Cultured Cells DNA kit | AS1620 |  | Promega, Madison, WI, USA |
| Fast Bisulfite Conversion Kit | ab117127 |  | Abcam, Cambridge, UK |
| EpiScope® MSP Kit | R100A |  | Takara Bio, Shiga, Japan |
